# How many water molecules are needed to solvate one?†

**DOI:** 10.1039/d0sc05785a

**Published:** 2020-12-18

**Authors:** Alessandro Rognoni, Riccardo Conte, Michele Ceotto

**Affiliations:** Dipartimento di Chimica, Università degli Studi di Milano Via Golgi 19 20133 Milano Italy michele.ceotto@unimi.it

## Abstract

Many efforts undertaken to study the solvation process have led to general theories that may describe mean properties, but are unable to provide a detailed understanding at the molecular level. Remarkably, the basic question of how many solvent molecules are necessary to solvate one solute molecule is still open. By exploring several water aggregates of increasing complexity, in this contribution we employ semiclassical spectroscopy to determine on quantum dynamical grounds the minimal network of surrounding water molecules to make the central one display the same vibrational features of liquid water. We find out that double-acceptor double-donor tetrahedral coordination constituting the standard picture is necessary but not sufficient, and that particular care must be reserved for the quantum description of the combination band due to the coupling of the central monomer bending mode with network librations. It is actually our ability to investigate the combination band with a quantum-derived approach that allows us to answer the titular question. The minimal structure eventually responsible for proper solvation is made of a total of 21 water molecules and includes two complete solvation shells, of which the whole first one is tetrahedrally coordinated to the central molecule.

## Abstract 2

How quantum spectroscopic simulations can explain water solvation by comparison with experimental spectra.

## Graphical abstract

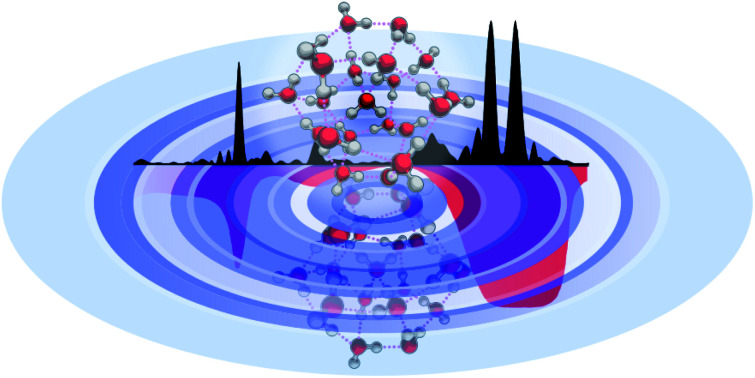

## 1. Introduction

The famous sorites paradox was first stated by Eubulides of Miletus in the 4th century B.C. In its original version it amounts to the question of down to which size a heap of sand can be still considered as such, when grains are removed one by one. A chemical-physical variant of the paradox can be formulated by counting how many water molecules make up the smallest water droplet, *i.e.* a supramolecular aggregate featuring the same properties as liquid water. Strictly related to this issue is the ubiquitous concept of solvation. Solvation is characterized by the nature of solute–solvent interactions, and often implies a structural reorganization of both. In the case of hydration, *i.e.* solvation performed by water, a specialized network of hydrogen bonds is built around the solute with solvent molecules arranged in shells. In practice, crucial phenomena like protein folding or reactivity in the condensed phase are strictly dependent on the mechanism of solvation. The structural organization of the solvent is also fundamental in explaining the hydrophobic force, sometimes described as the aggregation or desolvation of water repelling solutes that is thought to favor water-displacement equilibria when a ligand, such as a drug, approaches a receptor site in a hydrated protein. The principles of hydration are still debated with a constantly evolving consensus, which has moved over time from the original iceberg model to more recent and refined descriptions.^1^ Therefore, it is not an overstatement to affirm that the investigation of the spatial organization and number of water molecules needed to hydrate a central target species is of utmost importance and still an open issue.^2–6^

The conclusions that can be drawn from such a study certainly depend on the polarity and structural characteristics of the solute, but perhaps the first “solute” worth studying is water itself. In spite of the many attempts, the issue has not been settled yet. The reason for this lack of success is essentially two-fold. From the experimental point of view, it is difficult to determine with sufficient precision the number of molecules constituting a water cluster of medium-large size or to identify the specific cluster arrangement under investigation. Theoretically, it is hard to include key quantum effects in a simulation involving several dozens of degrees of freedom. To investigate the solvent at the molecular level one should neither employ rigid-molecule models within coarse-grained approaches nor invoke uniform medium approximations returning average properties. Conversely, each and every solvent molecule must be described explicitly and accurately according to the laws of quantum mechanics.

**Fig. 1:**
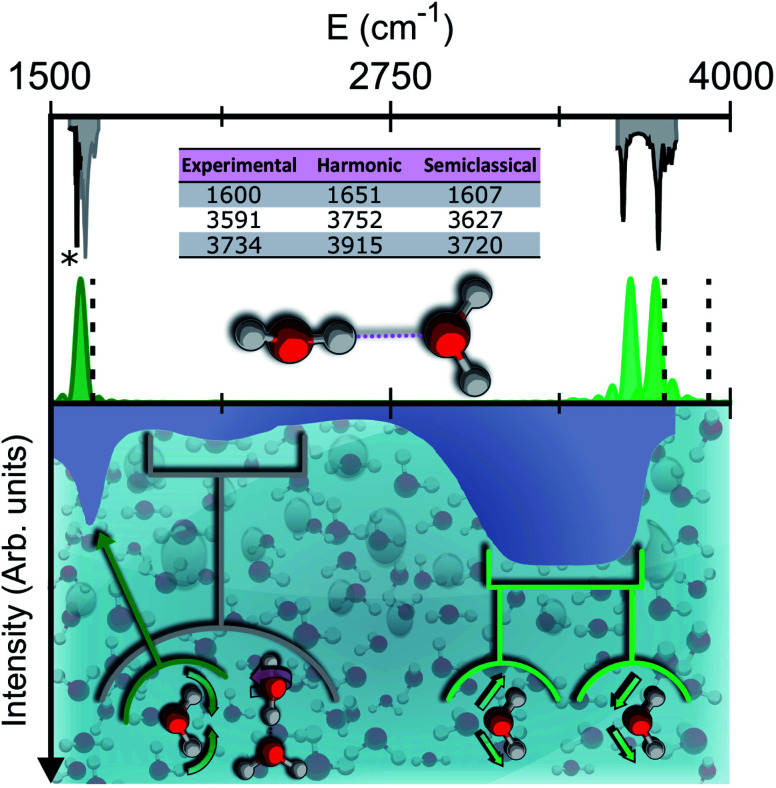
Semiclassical and experimental spectra of the water dimer. Top half: comparison between the experimentally detected fundamental frequencies of vibration for the water dimer (grey)^9–11^ and their theoretical semiclassical counterpart (green). The dashed vertical lines locate the harmonic estimates. The asterisk helps the eye detect the exact position of the reference experimental bending signal. Bottom half: the experimental IR spectrum of liquid water (solid contour). The normal modes involved in several signals are indicated with arrows and associated with the corresponding portions of the spectrum.

Vibrational spectroscopy provides a powerful experimental and theoretical tool to get insights into the molecular structure.^7^ In liquid water, the infrared (IR) spectrum is characterized by a broad band for the stretches, located between 3200 and 3550 cm^−1^, which is strongly red shifted with respect to the corresponding lines of the isolated water molecule at 3657 and 3756 wavenumbers.^8^ This is determined by the unique and complex network of intermolecular hydrogen bonds which weakens the intramolecular stretches. Other relevant spectral features of liquid water include the band of hindered translations and rotations (librations) below 1000 cm^−1^, the bending signal (at around 1650 cm^−1^), and the combination band of librations and bendings spanning the region around 2100 cm^−1^. The features of the liquid water IR spectrum are shown in the bottom half of Fig. 1.

We stipulate that for a water molecule to be labeled ‘solvated’ it must possess spectral features that resemble those of the bulk. In particular, besides the intramolecular bending and stretching signals, it is also crucial that the combination band of bending with librations matches the corresponding band of the bulk spectrum. A simple description of bulk liquid water depicts the H_2_O molecules as tetrahedrally coordinated, which means that each individual acts as a double donor and double acceptor of hydrogen bonds. Reality is more complex, because liquid water is characterized by a variety of hydrogen-bond lengths and coordination numbers.^12,13^ For instance, it is possible to find locally overcoordinated subunits, featuring three-acceptor two-donor configurations, which are responsible for the spectral tails at high frequency (on the acceptor side) and low frequency (on the donor side) of the stretching band.^14^ Tri-, tetra-, and penta-coordinations are presumably the most common instances, and by means of Raman multivariate curve resolution spectroscopy (MCR-Raman), it has been possible to identify the contribution of tetra-coordinated molecules within the wide stretching band of liquid water.^12^ Another powerful investigation tool, THz spectroscopy, covers instead the typical energy range of intermolecular interaction in condensed phases. It is able to characterize the collective motion in liquids and has permitted spectroscopic signals at 200 cm^−1^ and 80 cm^−1^ to be assigned to first and second solvation shell dynamics, respectively.^15^

The study of water clusters of increasing size provides a way to get molecular level insights into condensed phase systems like liquid water and ice.^16^ Many experimental and theoretical investigations have been undertaken to describe the intermolecular interactions of these systems, going from small quasi-planar (with reference to the oxygen atoms) clusters to larger ones.^17–20^ On the theoretical side, this has led to the construction of accurate potential energy surfaces (PESs),^21,22^ search for geometric and energy minima in a very complicated energy landscape, and spectroscopic calculations.^23,24^ For instance, the structural characteristics of water clusters beyond the transition between “all-surface” and “interior” ones have been studied. The transition takes place at (H_2_O)_17_ when one monomer is included in the framework built by the ensemble of the other water molecules.^25^ Another remarkable and very recent study, aimed at assessing the minimum system size showing bulk-like properties, has been performed on ice I.^26^ The main target of that investigation was to find the minimum dimensionality needed by a water cluster to show the hydrogen-bond pattern of ice I. Experimental studies and classical molecular dynamics simulations concerning infrared spectra and free energy estimates have been weighed in to conclude that in the range between 90 and 150 water molecules ice I is present in a mixture with amorphous clusters. In spite of such a variety of research efforts, some important aspects have not yet been sorted out and are still controversial. For instance, is tetrahedral coordination strictly necessary for a target water molecule to display the spectral properties of bulk liquid water? Is it sufficient? And, finally, what is the minimum number of water molecules required to fully solvate a single one?

## 2. Results and discussion

To give a precise answer to these questions we performed a quantum investigation by means of semiclassical spectroscopy, a theoretical and computational technique suitable for dealing with high dimensional or complex systems and reproducing quantum effects starting from classical trajectories.^27–31^ The upper panel of Fig. 1 demonstrates the high accuracy of SC spectroscopy in an application to the water dimer when compared with experimental results. However, it also shows that, as expected, the spectroscopic features of the water dimer (especially at high frequency) are not compatible with those of bulk water, confirming that more elaborate structures are necessary to mimic the bulk. Semiclassical methods have been applied successfully in a range going from small molecules,^32–34^ including fluxional ones,^35^ to medium-large systems.^36^ For this work we employed the multiple coherent states divide-and-conquer semiclassical initial value representation technique (MC-DC SCIVR),^37^ which has permitted open issues concerning protonated glycine supramolecular systems to be solved^38^ and has already been applied to the vibrational investigation of small water clusters.^39^ The interested reader may find more theoretical information on semiclassical dynamics and spectroscopy in Section 1 of the ESI.† For our semiclassical calculations we employed the water many-body MB-pol potential energy surface.^40^ We ran single-trajectory simulations about 0.7 ps long with a time step of about 0.12 fs. The initial energy of vibration was set equal to zero for the low-frequency modes (those with harmonic frequencies below the bending frequency), while the rotational and the translational motion of each monomer were eliminated at each step and the velocities were rescaled accordingly. More details to permit the reproducibility of results are provided in Section 2 of the ESI.†

**Fig. 2:**
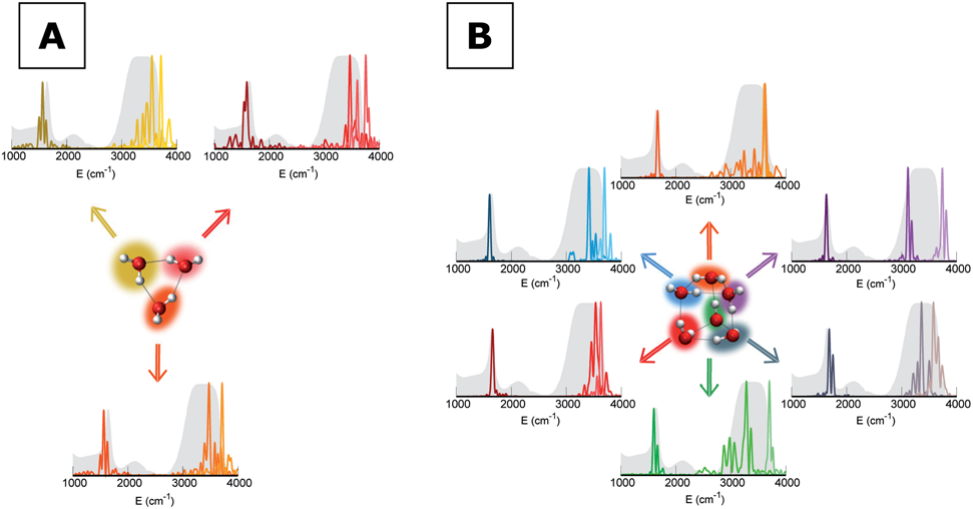
Power spectra for bending and stretches of individual monomers of small water clusters. (A) Trimer and (B) hexamer prism. The colored haloes link each monomer to the appropriate power spectrum. Color nuances are darker for bendings and lighter for symmetric and asymmetric stretches. The IR spectrum of liquid water is reported as shaded gray areas. The intensities of power spectra have been scaled to match the maximum of the corresponding experimental IR band.

Our analysis starts by studying three small water clusters: the dimer, which we have already anticipated; the trimer; the hexamer, this one in its prism configuration. The remarkable information collected from these simulations is that the frequencies of asymmetric stretches are blue shifted with respect to the experimental band of liquid water, in spite of the wide range it spans, and the bending signal is shifted from its experimental counterpart too. These spectral features demonstrate that solvation is not achieved for this set of small clusters, as confirmed by Fig. 2, which reports for the trimer and the hexamer prism the power spectra obtained for the bending and stretches of each water monomer superimposed on the IR spectrum of liquid water. Detailed frequency values can be found in Section 3 of the ESI.† We remark that the height of our peaks is not related to the experimental IR intensity (see Section 1 of the ESI).† Therefore, we have chosen to scale the intensities to ease the comparison between theory and experiments. From a structural point of view, the monomers in these small clusters are not tetrahedrally coordinated, hinting at the necessity to have an appropriate shell of coordination in order to display solvation properties, as suggested by Raman and THz spectroscopy studies. Fig. 2 also points out a key characteristic of calculated power spectra. They are able to reproduce all vibrational energy levels including those not involved in active IR or Raman transitions. For this reason our semiclassical simulations provide plenty of spectroscopic information and incorporate not only the target signals for bending and stretch fundamentals, but also combined excitations, Fermi resonances, overtones, and secondary signals related to other vibrational modes strongly coupled to the target ones. It is indeed the high density of vibrational states in association with inter-monomer interactions that shapes the wide IR bands typical of liquid water, an aspect also confirmed experimentally.^41,42^

**Fig. 3:**
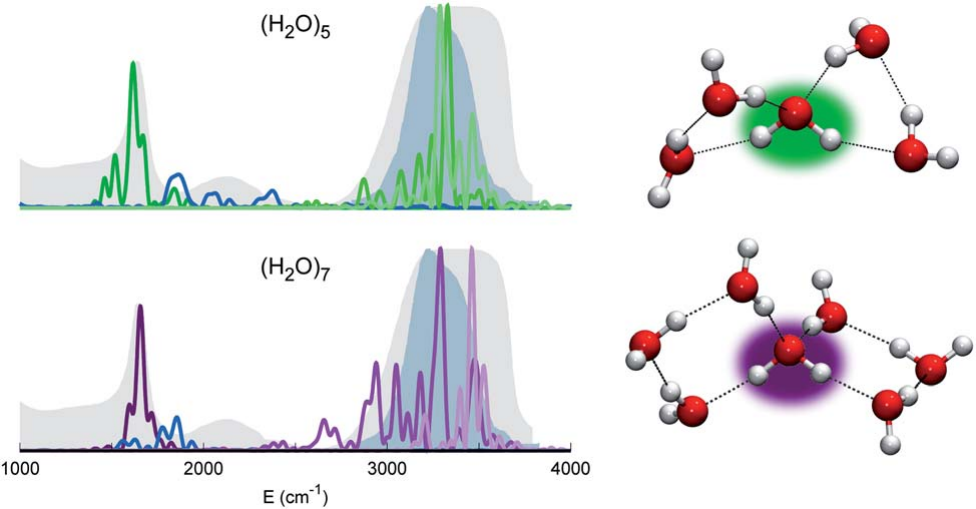
Power spectra of the tetrahedrally coordinated monomer in different water clusters. Top left column: pentamer; bottom left column: heptamer. The combined excitation of the bending with the most coupled libration mode is also reported in dark blue. The liquid water IR spectrum is shown as shaded gray areas, and the MCR-Raman band (from ref. 12) for the stretches of tetrahedrally coordinated monomers in liquid water (light blue) is also presented.

Before moving to more complex systems, we note that there are two water oligomers that do have (in one of their several isomers) tetrahedral coordination around one of the monomers. They are the water pentamer and heptamer, for which the target stretches are well inside the corresponding IR band of liquid water (see Fig. 3). However, this does not constitute sufficient evidence of solvation. In fact, the IR spectrum of liquid water is also characterized by the presence of a band in the 1900–2300 cm^−1^ range due to combined excitations of intra-monomer bendings and inter-monomer librations, as mentioned above. This feature can be explored by exploiting the capability of semiclassical spectroscopy to recover quantum effects. Specifically, the signal of the combined excitation of the bending of the target monomer with the libration most coupled to it (according to the analysis of the Hessian matrix at the equilibrium configuration) has been simulated showing that it is located outside the corresponding band in liquid water. This proves that even the presence of a 4-monomer coordination shell is not sufficient to achieve solvation, and an investigation at the quantum level able to reproduce combination bands is fundamental to point out this aspect. Furthermore, for tetrahedrally coordinated water molecules we can employ the MCR-Raman spectrum presented in ref. 12 to narrow the stretching band of liquid water. In Fig. 3, the MCR-Raman spectrum has been highlighted.

We note that, while the pentamer with its unperturbed tetrahedral coordination has both stretches well within the band, the asymmetric stretch for the heptamer is displaced toward the border of it. This is interpreted as the effect due to two additional monomers not directly coordinated to the central molecule, a first clear indication that tetrahedral coordination is not sufficient, and that a more extended network is required to reproduce the solvation features.

**Fig. 4:**
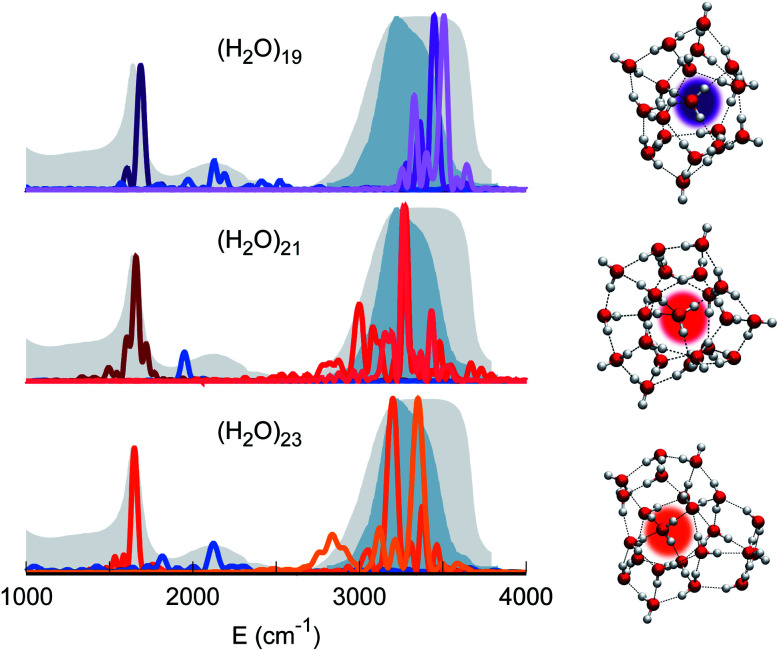
Power spectra of the target monomer for larger clusters. The peaks corresponding to bendings, stretches, and combined bending-libration bands of (H_2_O)_19_ (top), (H_2_O)_21_ (middle), and (H_2_O)_23_ (bottom) are reported.

Therefore, our quest for the minimal solvation structure proceeds by investigating more sizable systems for which a double shell of coordination around a central, target monomer is present. This is the case, for instance, of (H_2_O)_19_. The spectral features of the central monomer of this aggregate are shown in Fig. 4. Even if the results are closing in toward the expected spectroscopic characteristics of a solvated molecule, some inconsistencies are still present. The bending signal is shifted from the corresponding band in liquid water, and the stretches, even if located at still reasonable frequencies, are quite blue shifted with respect to the tetrahedral coordination band. We reckon that another, more complex structure needs to be studied and move to (H_2_O)_21_. In this case, the spectroscopic features of the bending and stretches of the target monomer are exactly where they are expected to be for a solvated water molecule. The final check is given by the combination peak, which is located at 1950 cm^−1^, *i.e.* still within the range of the IR experimental combination band. For these reasons our guess is that (H_2_O)_21_ represents the smallest water aggregate in which one monomer is solvated in the same way as molecules in the liquid.

To confirm this hypothesis we also analyzed a bigger structure, *i.e.* (H_2_O)_23_. In this case, even the combination signal moves to the center of the liquid water band, confirming that a solvation structure has indeed been reached. This shift is due to the higher frequency of the libration mode most coupled to the target bending (about 240 cm^−1^ at the harmonic level) in the case of (H_2_O)_23_ with respect to (H_2_O)_21_. In the (H_2_O)_23_ cluster, similarly to the heptamer but in a less prominent way, the asymmetric signal moves toward the edge of the tetrahedral coordination band revealing that, even if solvation is maintained, the two additional monomers have slightly influenced the coordination and solvation network. Detailed results can be found in Section 3 of the ESI.†

**Fig. 5:**
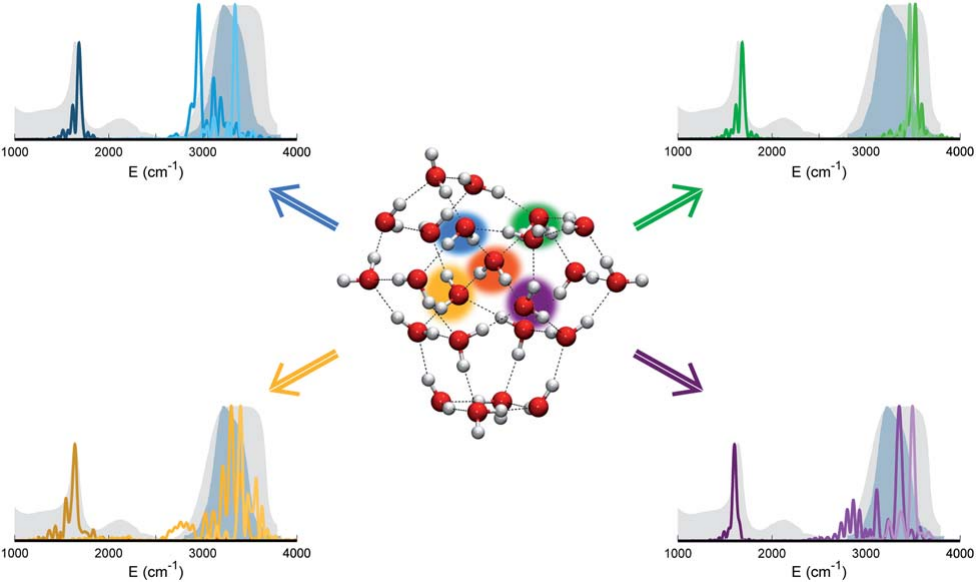
Power spectra of bending and stretch signals for the four monomers that are hydrogen bonded to the central monomer in (H_2_O)_23_. Different monomers and the corresponding power spectra are indicated with different colors (blue, green, violet, and yellow). The central monomer is highlighted in orange. Despite the tetrahedral coordination of these monomers, none of them matches the liquid water experimental spectra.

The persistent influence of outer additional monomers triggered our interest in having a closer look at the structural properties of (H_2_O)_23_. In particular, Fig. 5 focuses on the four monomers constituting the first coordination shell for the central one. Each of these four water molecules is itself tetrahedrally coordinated, but several spectral features do not match the liquid water ones. This is further confirmation that tetrahedral coordination alone does not warrant solvation, and that it is necessary to account for the overall network of hydrogen interactions which provides a complex and cooperative way for solvation in water.

## 3. Conclusions

Even though the bands characterizing the IR spectrum of liquid water are necessarily broad, a comparison with the results of accurate quantum spectroscopic simulations is able to discriminate the effective solvation properties of a central molecule embedded in clusters of increasing size. Small water clusters like the dimer, trimer, and hexamer are spectroscopically incompatible with solvation and their structure obviously lacks an appropriate coordination shell, which is found instead in the pentamer and heptamer that display tetracoordination at the central molecule. However, even in these latter oligomers the target spectral features do not match those of liquid water with reference to the combination band of bending and librations. Eventually, the size of the water aggregate had to be raised to 21 monomers in order to find the first example of a structure providing a proper solvation of its central molecule. This finding was confirmed by the study of a 23-monomer cluster. The bottom line is that tetrahedral coordination is a necessary but not sufficient condition; the minimal solvation structure, the primary aim of our quest, is found to consist of a central water molecule surrounded by at least 20 water monomers organized in a double coordination shell. Substantial cooperative effects through the extended bonding manifold are at work in determining the spectral properties of water solvation.

## Conflicts of interest

There are no conflicts to declare.

## Supplementary Material

SC-012-D0SC05785A-s001
