# Supplementary material for: How many water molecules are needed to solvate one?
Source: Chem Sci. 2020 Dec 18;12(6):2060–4. doi: 10.1039/d0sc05785a (PMC8179311; doi:10.1039/d0sc05785a)
Supplement: SC-012-D0SC05785A-s001 [file SC-012-D0SC05785A-s001.pdf]

Electronic Supplementary Material (ESI) for Chemical Science.
This journal is © The Royal Society of Chemistry 2020

Supplementary Information for

How many water molecules are needed to solvate one?

Alessandro Rognoni, Riccardo Conte, and Michele Ceotto*

E-mail: michele.ceotto@unimi.it

This PDF file includes:

Materials and Methods

Figs. S1 to S6

Tables S1 to S7

References

Contents

1 Theory **3**

1.1 Semiclassical spectroscopy: A powerful quantum investigation technique . . . 3

2 Methodological approach **8**

2.1 Assigning normal modes to specific water monomers 8

2.2 Combination band calculation 12

3 Benchmark calculations **14**

3.1 Vibrational frequencies of small-dimensional water clusters 14

3.2 Vibrational frequencies of tetrahedrally coordinated water molecules 17

References **20**

1 Theory

1.1 Semiclassical spectroscopy: A powerful quantum investigation technique

In molecular spectroscopy quantum aspects like zero-point energies, tunneling splittings, overtones, and combined excitations are of great importance. Purely quantum investigations are doable for small systems, but they become quickly computationally unaffordable as the complexity of the system under study increases. In spite of their intrinsic limitations and inaccuracies, *ad hoc* scaled harmonic approaches and classical investigations are usually undertaken in absence of a quantum treatment[1,2]. Semiclassical (SC) spectroscopy is a recently developed technique able to combine the advantages of classical and quantum spectroscopy into a novel and powerful investigation tool[3]. It has already been adopted to study the vibrational features of large molecules like fullerene[4], and biomolecules like glycine, nucleobases, and dipeptide derivatives[5–7]. Furthermore, SC spectroscopy has permitted to solve open issues related to supra-molecular systems. This is the case of some peculiar vibrational features of the glycine dimer and H_2-tagged protonated glycine, which have been successfully explained by means of SC spectroscopy[8]. All these results and other recent investigations provide a solid background for the investigations presented in the main body of this manuscript[6,9–19].

The foundation of semiclassical spectroscopy lies in the possibility to get the eigenvalues of the vibrational Hamiltonian starting from the Fourier transform of the survival amplitude

$$I(E) \propto \int dt\, e^{iEt/\hbar} \langle \Psi_0 | \Psi(t) \rangle, \tag{1}$$

where $|\Psi_0\rangle$ is an arbitrary state and $I(E)$ is the density of vibrational states. If one describes this arbitrary state as a linear combination of the Hamiltonian eigenstates $|E_j\rangle$, i.e. $|\Psi_0\rangle =$

$\sum_j c_j|E_j\rangle$, then Eq. (1) can be recast as

$$I(E) = \sum_j |c_j|^2 \delta(E - E_j), \tag{2}$$

which clearly demonstrates that peaks in the plot of $I(E)$ are centered at the vibrational eigenvalues.

Eq. (1) requires to evolve the initial state $|\Psi_0\rangle$ for a time t. In SC dynamics this is done by means of the SC propagator, whose most popular version is known as the Herman-Kluk propagator[20]. The reference state evolved in time using the Herman-Kluk propagator takes the form:

$$|\Psi(t)\rangle = \frac{1}{(2\pi\hbar)^{N_{vib}}} \int\int d\mathbf{p}_0 d\mathbf{q}_0 \, C_t(\mathbf{p}_0, \mathbf{q}_0) \, e^{iS_t(\mathbf{p}_0,\mathbf{q}_0)/\hbar} \, \langle \mathbf{p}_0, \mathbf{q}_0|\Psi_0\rangle |\mathbf{p}_t, \mathbf{q}_t\rangle. \tag{3}$$

Semiclassical propagation according to Eq. (3) requires to run full (N_{vib})-dimensional classical trajectories for a total time t, usually of the order of one picosecond, starting from momenta and coordinates ($\mathbf{p}_0, \mathbf{q}_0$). For this reason the initial value representation (IVR) denomination is often used to describe the method[21]. The instantaneous classical action $S_t(\mathbf{p}_0, \mathbf{q}_0)$ and the complex-valued prefactor $C_t(\mathbf{p}_0, \mathbf{q}_0)$ are fingerprints of the original derivation of the SCIVR theory as stationary phase approximation to Feynman's path integral representation of the quantum propagator[22]. Finally, the $|\mathbf{p}_0, \mathbf{q}_0\rangle$ and $|\mathbf{p}_t, \mathbf{q}_t\rangle$ states are the initial and time-evolved basis set coherent states. It is this sophisticated mathematical machinery which permits the quantitative description of quantum effects starting from classical trajectories.

For application of SC dynamics to the evaluation of Eq. (1), Kaledin and Miller introduced their time-averaged formulation of the Herman-Kluk propagator and worked out the following SC expression for the spectral density

$$I(E) \propto \int\int d\mathbf{p}_0 d\mathbf{q}_0 \left| \int_0^T dt\, e^{i[S_t(\mathbf{p}_0,\mathbf{q}_0)+\phi_t(\mathbf{p}_0,\mathbf{q}_0)+Et]/\hbar} \langle \mathbf{p}_t,\mathbf{q}_t|\Psi_0\rangle \right|^2 , \tag{4}$$

where the total evolution time is indicated by T and only the phase of the prefactor $\phi_t(\mathbf{p}_0, \mathbf{q}_0)$ is employed. As anticipated, a plot of $I(E)$ versus E, i.e. the power spectrum of the vibrational Hamiltonian, allows one to identify the eigenvalues as the energies at which the several spectral signals are centered. The corresponding frequencies of vibration are readily obtained by difference with respect to the ZPE value. Our power spectra provide a quantum comparison to experimental IR and Raman spectra regarding the vibrational frequencies, a feature which is at the heart of the investigations described in the main body of the manuscript.

Eq. (4) requires a phase space integration which is computationally unaffordable as the system dimensionality increases beyond a few degrees of freedom. In this case it is necessary to rely on a SC technique able to provide accurate vibrational frequencies from a single or few trajectories. The multiple coherent states semiclassical approach (MC SCIVR) makes this possible[9]. It is based on the fact that quantum eigenvalues can be estimated exactly by a single-trajectory SC simulation, provided that the classical trajectory is run at that exact energy[23]. Clearly the exact energy is not known *a priori*, but the MC-SCIVR technique provides anyway a very accurate approximation to it. The approach adopts the same mathematical formalism of Eq. (4) but relies on the choice of tailored reference states able to enhance the spectral signal associated to the energy at which the simulation is performed. Furthermore, the MC SCIVR has opened the possibility to adopt direct *ab initio* molecular dynamics for SC spectroscopy with application to medium-large systems for which accurate analytical potential energy surfaces are not available.

Notwithstanding, application of SC spectroscopy to large systems has been hampered by the difficulty to get sensible spectroscopic signals when the quantum overlap in Eq. (4) is

not sizeable due to its multi-dimensional product nature. To overcome this major issue, very recently, the divide-and-conquer SC method (DC SCIVR) has been introduced[4,15]. The working equation of DC SCIVR is similar to Eq. (4) but based on the projection of the several dynamical quantities onto lower dimensional subspaces appropriately chosen to accommodate strongly coupled vibrational modes

$$\tilde{I}(E) \propto \int\int d\tilde{\mathbf{p}}_0 d\tilde{\mathbf{q}}_0 \left| \int_0^T dt\, e^{i[\tilde{S}_t(\tilde{\mathbf{p}}_0,\tilde{\mathbf{q}}_0)+\tilde{\phi}_t(\tilde{\mathbf{p}}_0,\tilde{\mathbf{q}}_0)+Et]/\hbar} \langle \tilde{\mathbf{p}}_t, \tilde{\mathbf{q}}_t | \tilde{\Psi}_0 \rangle \right|^2 . \quad (5)$$

The total power spectrum is eventually reconstructed as a convolution of the lower dimensional spectra, and the tilde symbol (~) has been added to those quantities that have been projected onto a M-dimensional subspace. All the quantities appearing in Eq. (5) can be straightforwardly projected, with the exception of the action. This happens because the potential V of the systems under investigation is not separable (it cannot be written down exactly as a sum of terms coming from different subspaces). To project the action we adopted the following equation:

$$\tilde{S}_t(\tilde{\boldsymbol{p}}_0, \tilde{\boldsymbol{q}}_0) = \int_0^t dt' \left[\frac{1}{2} \tilde{\boldsymbol{p}_{t'}}^T \tilde{\boldsymbol{p}}_{t'} - \left(V(\tilde{\boldsymbol{q}}_{t'}, \boldsymbol{q}_{t'}^{(N_{vib}-M)}) - V(\tilde{\boldsymbol{q}}_{eq}, \boldsymbol{q}_{t'}^{(N_{vib}-M)}) \right) \right] , \quad (6)$$

which is exact for separable systems and approximates the projected action for non-separable ones. The multiple coherent and divide-and-conquer techniques can be interfaced leading to single-trajectory simulations for subspaces of reduced dimensionality. The technique is called multiple coherent states divide-and-conquer semiclassical initial value representation (MC-DC SCIVR), which is indeed the kind of approach adopted for the investigations presented in the main body of the manuscript. The key observation that allows one to reduce the number of trajectories is that most of the contribution to the spectrum comes from the trajectories having an energy as close as possible to the true but unknown quantum mechanical eigenvalues. On

this basis, in the multiple coherent framework proper initial conditions and tailored reference states are employed to decrease the number of trajectories down to one per simulation.

For any target state α (like the ground state or excited states), the reference state $|\tilde{\Psi}_0\rangle$ can be chosen as a combination of coherent states

$$|\tilde{\Psi}_0\rangle_\alpha = \prod_{k=1}^{M} |\tilde{p}_k^{eq}, \tilde{q}_k^{eq}\rangle + \varepsilon_{k,\alpha}| - \tilde{p}_k^{eq}, \tilde{q}_k^{eq}\rangle. \tag{7}$$

In the previous equation, $\tilde{\mathbf{q}}^{eq}$ are the equilibrium positions and $\tilde{\mathbf{p}}^{eq}$ the harmonically approximated mass-scaled momenta

$$\tilde{p}_k^{eq} = \sqrt{\hbar\omega_k(2n_k + 1)}, \tag{8}$$

where ω_k is the harmonic frequency of the k-th mode and n_k its vibrational quantum number. In Eq. (7), $\varepsilon_{k,\alpha}$ is the k-th component of an M-dimensional array of integers appropriately chosen to enhance the spectral signal associated to the specific state α. For instance, a collection of $\epsilon_{k,\alpha} = +1$ values allows one to get the ZPE signal. As another example, if only the k'-th element of the array is equal to -1 (with all other array elements equal +1), then the spectral features corresponding to α states that in harmonic approximation have an odd number of quanta in the k'-th degree of freedom are emphasized. The procedure is rigorous in the case of uncoupled harmonic oscillators, and only approximated but still very helpful in the general case, in which less intense signals due to states strongly coupled to the α one may also be present.

2 Methodological approach

2.1 Assigning normal modes to specific water monomers

In water clusters, the normal modes are strongly delocalized over different water molecules. So, the assignment of a vibrational mode to a specific monomer must be done carefully. For assigning the normal modes to specific water molecules, we relied on the time-averaged root-mean-square displacement (TA RMSD). Specifically, we considered one vibrational mode at a time, leaving the others at their equilibrium values. Along the trajectory of the select mode, we summed up the deviation from the equilibrium positions of all the water molecules that compose the cluster. At the end of the trajectory, we assigned the vibrational normal mode to the water molecule with the highest TA RMSD. The underlying principle is that if a normal mode primarily contributes to the motion of a specific monomer, then it will be assigned to that water molecule. This approach corresponds to the usual chemical interpretation of the assignment procedure. Table 1 reports the values of TA RMSD of the central water molecule of $(H_2O)_{21}$ obtained for all the bendings and stretches. The three normal modes assigned to the central water molecule are q_{132} (bending), q_{149}, and q_{153} (stretches). Fig. 1 shows the behavior of TA RMSD with respect to the central water molecule of the three select normal modes.

The distinction between symmetric and asymmetric stretches is a more complex task because the same normal mode can act with a symmetric character on a water molecule but with an asymmetric character on another one. To distinguish the symmetric stretches from the asymmetric ones, we adopted an approach based on a reward function. For each stretching mode and at each time-step of the trajectory, a reward of +1 is assigned to a monomer if the two OH distances change in the same direction, *i.e.* if the stretch is symmetric. In the same fashion, a -1 reward is assigned if the OH distances change in opposite directions, the key feature of asymmetric stretch. It is noteworthy that we always obtained full (either positive or negative) rewards

for all the stretches of the clusters we studied. This means that even though the same normal mode can have different behavior when acting on different monomers, its pure symmetric or asymmetric character is preserved along the trajectory. In fig. 2 we showed the two types of reward plot we obtained for all the stretches studied in this work. Depending on the normal mode under consideration and on the water molecule it is attributed to, the reward is always positive (symmetric stretch) or always negative (asymmetric stretch). As anticipated earlier and in the main text, for all the simulations we employed a single classical trajectory, using the MC-DC SCIVR method developed by our group.

Table 1: **Values of TA RMSD with respect to the motion of the central water molecule obtained for all the bendings and stretches of the $(H_2O)_{21}$ cluster.** The normal modes "q" are labeled according to their harmonic frequencies. It is possible to observe that the three normal modes 132, 149, and 153 (one bending and two stretches) can be unambiguously assigned to the central water molecule of the cluster.

q	TA RMSD/au		q	TA RMSD/au	
**132**	1.24	10^{-1}	**156**	8.22	10^{-3}
**149**	9.13	10^{-2}	**134**	8.17	⋮
**153**	9.11	⋮	**141**	7.05	
**137**	8.66		**167**	6.63	
**154**	6.99		**129**	6.16	
**130**	6.37		**144**	4.75	
**122**	5.82		**145**	4.68	
**131**	4.08		**171**	4.09	
**150**	3.61		**125**	4.03	
**168**	2.90		**161**	3.40	
**124**	2.48		**163**	2.99	
**151**	2.44		**147**	2.12	
**158**	2.35		**165**	2.01	
**157**	2.25		**142**	1.89	
**138**	2.21		**175**	1.79	
**126**	2.19		**143**	1.52	
**160**	2.15		**172**	1.22	
**139**	2.09		**146**	1.19	
**133**	2.01		**123**	1.16	
**159**	1.92		**170**	1.05	
**166**	1.88		**179**	9.17	10^{-4}
**155**	1.86		**164**	8.23	⋮
**152**	1.73		**181**	7.68	
**136**	1.39		**178**	3.96	
**128**	1.38		**174**	3.91	
**121**	1.33		**173**	3.31	
**162**	1.19		**176**	3.12	
**135**	1.07		**177**	2.81	
**169**	9.75	10^{-3}	**182**	2.31	
**127**	9.75	⋮	**183**	1.99	
**140**	9.00		**180**	1.93	
**148**	8.24				

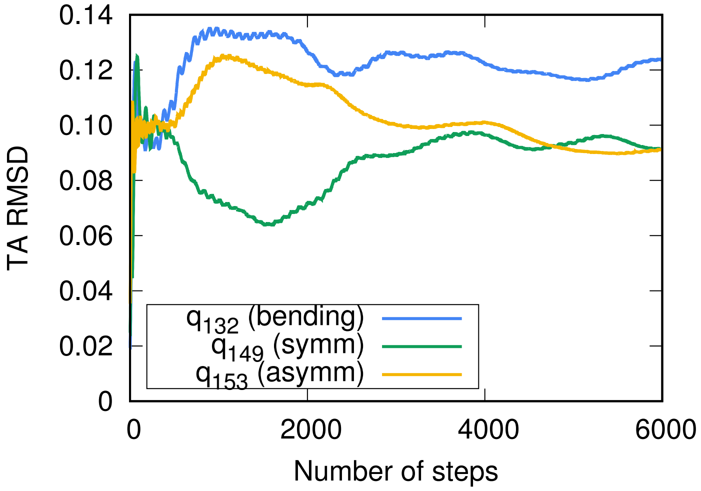

Figure 1: **Behavior of TA RMSD for the normal modes assigned to the central monomer of the $(H_2O)_{21}$ cluster. q_{132}: bending; q_{149}: symmetric stretch; q_{153}: asymmetric stretch.**

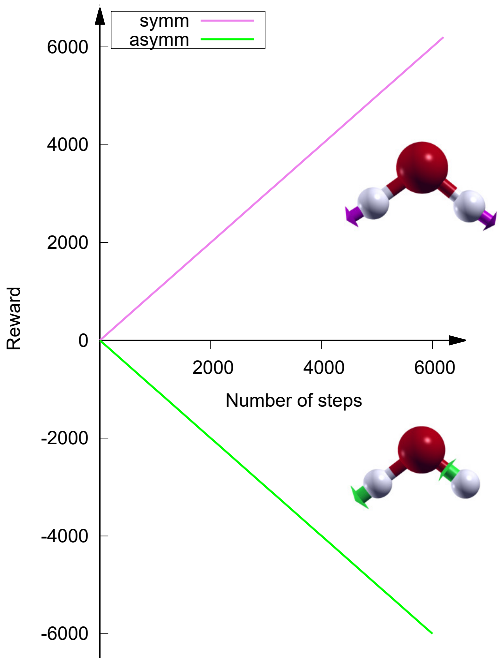

Figure 2: **Rewards obtained for all the stretches analyzed in this work.** Violet: reward assigned to symmetric stretches. Green: same but referred to asymmetric stretches. Our simulations are made of 6000 steps, so it is clear that we always obtain full (positive or negative) rewards.

2.2 Combination band calculation

Once the bending associated to central monomer has been assigned, it is possible to calculate the band associated to the combined excitation of the bending with all the low-frequency librational modes. This band would span a very large portion of the spectrum because, for a general $(H_2O)_n$ cluster, there are $6(n-1)$ low-frequency vibrational normal modes, and hence $6(n-1)$ combination signals. To obtain results comparable between the several water aggregates investigated, we chose to focus on the libration that exhibits the highest coupling with the bending of the central water molecule.

The challenge now is how to recognize this special librational mode. First of all, a ZPE trajectory is run (a 0.7 ps-long trajectory with a timestep of 0.12 fs), without removing the angular momentum of the water molecules from the trajectory. In fact, the low-frequency modes are usually characterized by hindered rotations or translations, which could not be accurately described using the same approach employed for the intramolecular mode simulations. Along this trajectory, we apply the Hessian method developed by our group to identify the librational mode showing the highest coupling with the bending. In practice, the off-diagonal elements of the Hessian matrix, which correspond to the second derivative of the potential with respect to the bending and to the different librations, are calculated and their absolute values are summed up. In this way, we obtained time-averaged Hessian matrix elements, which are a direct measure of the coupling between normal modes. Specifically, the off-diagonal elements of the Hessian are equal to zero in the case of completely uncoupled oscillators. So, the magnitude of the mixed derivative of the potential with respect to two normal modes is a measure of their coupling. Therefore, the libration experiencing the highest coupling with the bending of the central monomer will be the one with the highest off-diagonal element of the time-averaged Hessian matrix. Fig. 3 shows the chosen libration for the $(H_2O)_{21}$ cluster. In fig. 4 we reported the behavior of the time-averaged off-diagonal elements of the Hessian matrix for different librational

modes. Once the target libration has been determined, it is possible to calculate the combination signal through a proper choice of the wavepacket. In this case, both the ϵ coefficients of Eq. (7) associated to the target bending and libration are set equal to -1, while leaving all the others equal to 0.

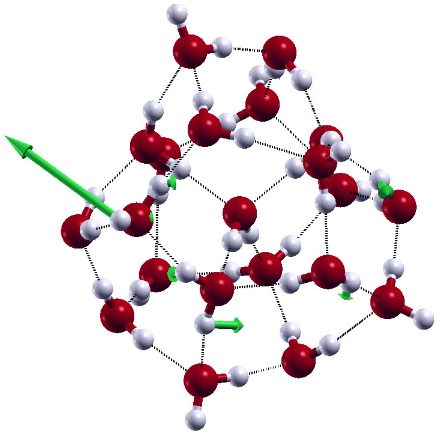

Figure 3: **Selected libration for the $(H_2O)_{21}$ cluster**. This normal mode is labeled q_{53} in Fig. 4.

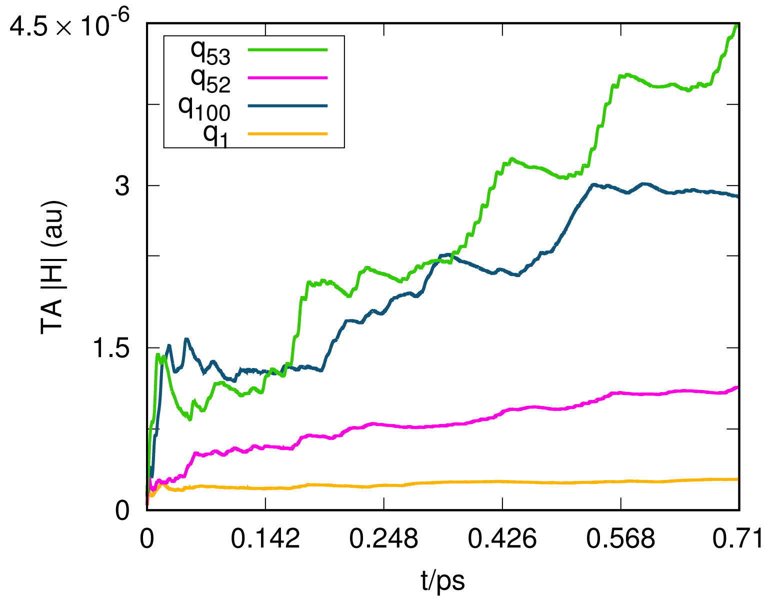

Figure 4: **Time-averaged absolute values of the Hessian matrix for the bending of the central monomer of $(H_2O)_{21}$.** The time-averaged matrix elements are compared for different librational motions: q_{53} is the select libration (reported in Fig. 3), q_{52} is a libration with similar harmonic frequency, q_1 is a libration with lower harmonic frequency, and q_{100} is characterized by a higher harmonic frequency.

3 Benchmark calculations

3.1 Vibrational frequencies of small-dimensional water clusters

Here we report the frequency values obtained with the MC-DC-SCIVR approach and discussed in the main text. The results are benchmarked against experiments and MultiMode (MM) or Local Monomer Model (LMM) calculations. Both the MM and the LMM simulations were carried out on a different potential energy surface (the WHBB one)[24]. Nonetheless, these values provide an accurate qualitative benchmark to confirm the accuracy of the MC-DC-SCIVR method. The vibrational spectrum of the water dimer is depicted in Fig. 5 and the corresponding frequencies are reported in Table 2. The vibrational frequencies of the water trimer and of the

water hexamer prism (Fig. 2 in the main text) are listed in Table 3 and in Table 4, respectively.

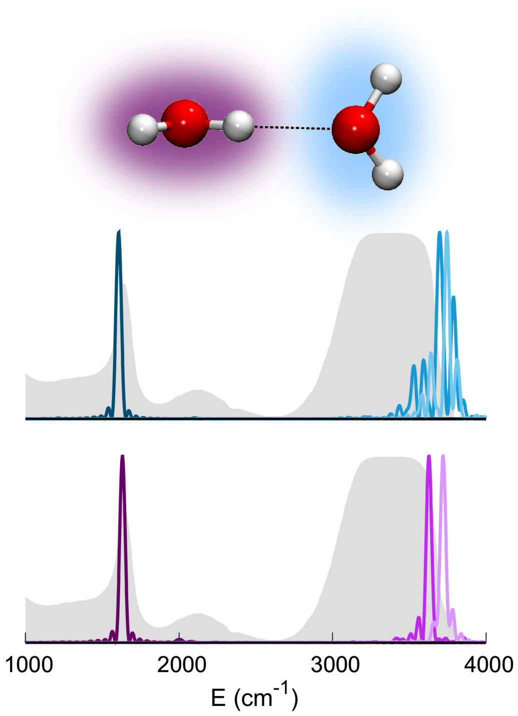

Figure 5: **Vibrational spectrum of the water dimer.** Peak colors are chosen in agreement with the water monomer they belong to. The liquid water infrared spectrum is reported as shaded gray areas.

Table 2: **Frequencies of the water dimer (cm^{-1}).** Column "q" reports the normal modes ordered according to their harmonic frequencies (Harm); column "MM" shows the MultiMode estimates; column "MC-DC SCIVR" reports the semiclassical frequencies obtained in the present work; under column "Exp" we have reported the experimental frequencies[25]. Mean absolute errors with respect to experiments (MAE exp) and MM calculations (MAE MM) are shown. The spectrum can be found in fig. 1 of the main text.

q	Exp	MM	MC-DC SCIVR	Harm
**7**	1600	1588	1607	1651
**8**	1617	1603	1632	1665
**9**	3591	3573	3627	3752
**10**	3661	3627	3698	3832
**11**	3734	3709	3720	3915
**12**	3750	3713	3745	3935
**MAE exp**	-	23	19	133
**MAE MM**	-	-	36	156

Table 3: **Frequencies of the water trimer (cm^{-1}). Labels are chosen in agreement with Table 2.**

q	MM	MC-DC SCIVR	Harm
**13**	1597	1562	1656
**14**	1600	1591	1661
**15**	1623	1561	1685
**16**	3486	3465	3636
**17**	3504	3550	3685
**18**	3514	3475	3694
**19**	3709	3753	3906
**20**	3716	3714	3915
**21**	3720	3714	3916
**MAE MM**	-	29	143

Table 4: **Frequencies of the water hexamer prism (cm^{-1}). Labels are chosen in agreement with Table 2. Under column "LMM" we have reported the Local Monomer Model estimates.**

q	LMM	MC-DC SCIVR	Harm
**31**	1606	1605	1652
**32**	1612	1600	1671
**33**	1620	1630	1680
**34**	1633	1659	1690
**35**	1654	1669	1711
**36**	1677	1682	1729
**37**	3092	3114	3307
**38**	3256	3283	3513
**39**	3372	3361	3603
**40**	3442	3412	3627
**41**	3482	3534	3729
**42**	3521	3622	3747
**43**	3579	3578	3789
**44**	3588	3627	3805
**45**	3630	3609	3818
**46**	3697	3739	3908
**47**	3706	3701	3910
**48**	3728	3694	3911
**MAE LMM**	-	25	161

3.2 Vibrational frequencies of tetrahedrally coordinated water molecules

In this section we report the frequencies associated to bendings, stretches and combination bands of water molecules with tetrahedral coordination. Table 5 reports the frequencies of the central water monomers of $(H_2O)_5$ and $(H_2O)_7$. Table 6 reports the vibrational frequencies of the central water molecule in larger water clusters. Specifically, Table 7 shows the vibrational frequencies of the monomers that make up the first solvation shell of the central water molecule of $(H_2O)_{23}$ (depicted in Fig. 6).

In Tables 5, 6, and 7, the vibrational frequencies of the target water molecules are high-

lighted if they are compatible with the experimental frequencies of liquid water. We remark that this comparison can be made only from a qualitative point of view. In fact, we can only rely on broad infrared signals to confirm the solvation of a target water molecule. Raman-MCR experiments somewhat help to narrow the stretching band in the case of tetrahedrally coordinated water molecules, but the signal still remains quite broad. At present, we can only give a qualitative description of a solvated water molecule. Any well-defined energy window that discriminates a solvated water molecule from a non-solvated one would not be neither accurate nor very helpful. More specifically, a plethora of stretching signals are compatible with a solvated water molecule within the experimental band, given the infinity number of geometries that it can assume.

Table 5: **Frequencies associated to the central monomers of small water clusters (cm^{-1}).** The corresponding MC-DC-SCIVR power spectra are reported in Fig. 3 of the main text. The MC-DC-SCIVR frequencies are reported under columns "SC", while the harmonic frequencies are listed under columns "Harm". Frequencies of bendings and combination bands that are comparable with the ones of the liquid water infrared spectrum as well as signals associated to symmetric and asymmetric stretches comparable with the MCR-Raman spectrum are highlighted.

	**$(H_2O)_5$**		**$(H_2O)_7$**	
	**SC**	**Harm**	**SC**	**Harm**
**bending**	**1616**	1662	**1656**	1680
**combination band**	1863	1902	1854	1909
**symmetric stretch**	**3329**	3600	**3290**	3469
**asymmetric stretch**	**3288**	3722	3461	3583

Table 6: **Frequencies associated to the central monomers of larger water clusters (cm^{-1}).** The corresponding MC-DC-SCIVR power spectra are reported in Fig. 4 of the main text. Labels are chosen in agreement with Table 5. Frequencies of bendings and combination bands that are comparable with the ones of the liquid water infrared spectrum as well as signals associated to symmetric and asymmetric stretches comparable with the MCR-Raman spectrum are highlighted.

	**$(H_2O)_{19}$**		**$(H_2O)_{21}$**		**$(H_2O)_{23}$**	
	**SC**	**Harm**	**SC**	**Harm**	**SC**	**Harm**
**bending**	1687	1696	**1659**	1697	**1649**	1709
**combination band**	**2127**	2313	**1950**	1980	**2124**	2233
**symmetric stretch**	3448	3607	**3276**	3462	**3200**	3425
**asymmetric stretch**	3506	3583	**3253**	3538	**3351**	3465

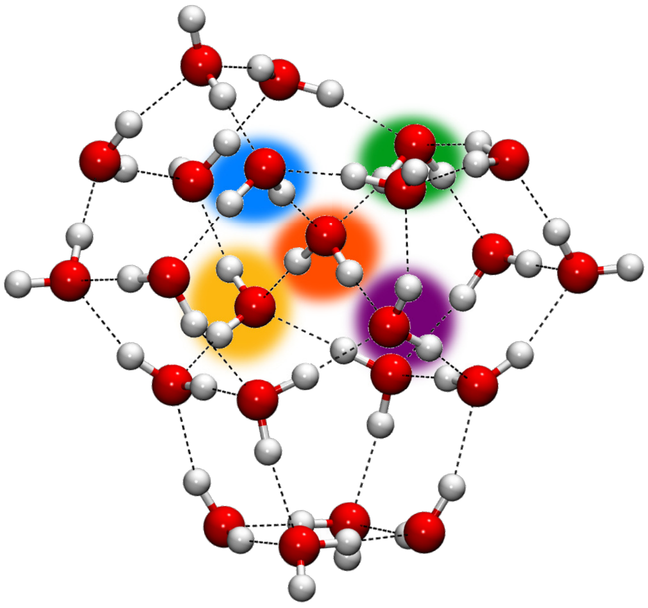

Figure 6: **Magnified view of the $(H_2O)_{23}$ cluster.** The monomers composing the first solvation shell of the central water molecule are highlighted with different colors (yellow, blue, green, and purple). The central water molecule is colored in orange.

Table 7: **MC-DC SCIVR frequencies associated to the monomers constituting the first coordination shell of the $(H_2O)_{23}$ cluster (cm^{-1}).** The colors of the labels are chosen to match the corresponding monomers in the previous figure. Frequencies of bendings and combination bands that match the IR spectrum of liquid water as well as signals associated to symmetric and asymmetric stretches matching the MCR-Raman spectrum are highlighted.

$(H_2O)_{23}$	Mon1	Mon2	Mon3	Mon4
**bending**	1684	**1640**	1602	1684
**symmetric stretch**	2961	**3303**	**3353**	3525
**asymmetric stretch**	**3343**	3402	3494	3465

References

(1) Scott, A. P.; Radom, L. Harmonic vibrational frequencies: an evaluation of Hartree-Fock, Møller- Plesset, quadratic configuration interaction, density functional theory, and semiempirical scale factors. *J. Phys. Chem.* **1996**, *100*, 16502–16513.

(2) Galimberti, D. R.; Milani, A.; Tommasini, M.; Castiglioni, C.; Gaigeot, M.-P. Combining static and dynamical approaches for infrared spectra calculations of gas phase molecules and clusters. *Journal of chemical theory and computation* **2017**, *13*, 3802–3813.

(3) Miller, W. H. Quantum dynamics of complex molecular systems. *Proc. Natl. Acad. Sci. USA* **2005**, *102*, 6660–6664.

(4) Ceotto, M.; Di Liberto, G.; Conte, R. Semiclassical "divide-and-conquer" method for spectroscopic calculations of high dimensional molecular systems. *Phys. Rev. Lett.* **2017**, *119*, 010401.

(5) Gabas, F.; Conte, R.; Ceotto, M. On-the-fly ab initio semiclassical calculation of glycine vibrational spectrum. *J. Chem. Theory Comput.* **2017**, *13*, 2378.

(6) Gabas, F.; Di Liberto, G.; Ceotto, M. Vibrational investigation of nucleobases by means of divide and conquer semiclassical dynamics. *J. Chem. Phys.* **2019**, *150*, 224107.

(7) Conte, R.; Gabas, F.; Botti, G.; Zhuang, Y.; Ceotto, M. Semiclassical vibrational spectroscopy with Hessian databases. *J. Chem. Phys.* **2019**, *150*, 244118.

(8) Gabas, F.; Di Liberto, G.; Conte, R.; Ceotto, M. Protonated glycine supramolecular systems: the need for quantum dynamics. *Chem. Sci.* **2018**, *9*, 7894–7901.

(9) Ceotto, M.; Atahan, S.; Tantardini, G. F.; Aspuru-Guzik, A. Multiple coherent states for first-principles semiclassical initial value representation molecular dynamics. *J. Chem. Phys.* **2009**, *130*, 234113.

(10) Ceotto, M.; Atahan, S.; Shim, S.; Tantardini, G. F.; Aspuru-Guzik, A. First-principles semiclassical initial value representation molecular dynamics. *Phys. Chem. Chem. Phys.* **2009**, *11*, 3861–3867.

(11) Ceotto, M.; Tantardini, G. F.; Aspuru-Guzik, A. Fighting the curse of dimensionality in first-principles semiclassical calculations: Non-local reference states for large number of dimensions. *J. Chem. Phys.* **2011**, *135*, 214108.

(12) Conte, R.; Aspuru-Guzik, A.; Ceotto, M. Reproducing deep tunneling splittings, resonances, and quantum frequencies in vibrational spectra from a handful of direct ab initio semiclassical trajectories. *J. Phys. Chem. Lett.* **2013**, *4*, 3407–3412.

(13) Di Liberto, G.; Ceotto, M. The importance of the pre-exponential factor in semiclassical molecular dynamics. *J. Chem. Phys.* **2016**, *145*, 144107.

(14) Micciarelli, M.; Conte, R.; Suarez, J.; Ceotto, M. Anharmonic vibrational eigenfunctions

and infrared spectra from semiclassical molecular dynamics. *J. Chem. Phys.* **2018**, *149*, 064115.

(15) Di Liberto, G.; Conte, R.; Ceotto, M. "Divide and conquer" semiclassical molecular dynamics: A practical method for spectroscopic calculations of high dimensional molecular systems. *J. Chem. Phys.* **2018**, *148*, 014307.

(16) Buchholz, M.; Grossmann, F.; Ceotto, M. Simplified approach to the mixed time-averaging semiclassical initial value representation for the calculation of dense vibrational spectra. *J. Chem. Phys.* **2018**, *148*, 114107.

(17) Bertaina, G.; Di Liberto, G.; Ceotto, M. Reduced rovibrational coupling Cartesian dynamics for semiclassical calculations: Application to the spectrum of the Zundel cation. *J. Chem. Phys.* **2019**, *151*, 114307.

(18) Conte, R.; Parma, L.; Aieta, C.; Rognoni, A.; Ceotto, M. Improved semiclassical dynamics through adiabatic switching trajectory sampling. *J. Chem. Phys.* **2019**, *151*, 214107.

(19) Micciarelli, M.; Gabas, F.; Conte, R.; Ceotto, M. An Effective Semiclassical Approach to IR Spectroscopy. *J. Chem. Phys.* **2019**, *150*, 184113.

(20) Herman, M. F.; Kluk, E. A semiclassical justification for the use of non-spreading wavepackets in dynamics calculations. *Chem. Phys.* **1984**, *91*, 27–34.

(21) Miller, W. H.; George, T. F. Semiclassical theory of electronic transitions in low energy atomic and molecular collisions involving several nuclear degrees of freedom. *J. Chem. Phys.* **1972**, *56*, 5637–5652.

(22) Feynman, R. P.; Hibbs, A. R. *Quantum mechanics and path integrals*; McGraw-Hill, 1965.

(23) De Leon, N.; Heller, E. J. Semiclassical quantization and extraction of eigenfunctions using arbitrary trajectories. *J. Chem. Phys.* **1983**, *78*, 4005–4017.

(24) Wang, Y.; Bowman, J. M. Ab initio potential and dipole moment surfaces for water. II. Local-monomer calculations of the infrared spectra of water clusters. *J. Chem. Phys.* **2011**, *134*, 154510.

(25) Bouteiller, Y.; Perchard, J. The vibrational spectrum of $(H_2O)_2$: Comparison between anharmonic ab initio calculations and neon matrix infrared data between 9000 and 90 cm^{-1}. *Chem. Phys.* **2004**, *305*, 1–12.
